# DHP23002 as a next generation oral paclitaxel formulation for pancreatic cancer therapy

**DOI:** 10.1371/journal.pone.0225095

**Published:** 2019-11-19

**Authors:** Eunseo Jang, Minhee Son, Junhee Jang, In-Hyun Lee, Sol Kim, Taejun Kwon, Yong-hyun Jeon, Woo-Suk Koh, Kil-Soo Kim, Sang Kyoon Kim

**Affiliations:** 1 Laboratory Animal Center, Daegu-Gyeongbuk Medical Innovation Foundation, Daegu, South Korea; 2 Dae Hwa Pharmaceutical Co. Ltd., Pangyo Research Laboratory, Sungnam City, South Korea; 3 College of Veterinary Medicine, Kyungpook National University, Daegu, South Korea; Virginia Commonwealth University, UNITED STATES

## Abstract

**Objective:**

This study aimed to develop a new oral paclitaxel formulation (DHP23002) and to evaluate its absorption and antitumor effects in a pancreatic tumor mouse model.

**Methods:**

To investigate the oral absorption of DHP23002, a newly developed lipid-based orally active paclitaxel formulation, a pharmacokinetic study of DHP23002, was conducted in mice (62.5 and 125 mg/kg). Moreover, to evaluate the antitumor effect of DHP23002 in pancreatic cancer treatment, the drug was administered to female athymic nude mice at 0 (vehicle), 25, 62.5, and 125 mg/kg on alternate days; the efficacy of the agent was compared with the efficacy of intravenous Taxol^®^ injections at 10 mg/kg once per week. After 3 weeks of administration, tumor growth in mice belonging to each group was further monitored for 4 weeks after discontinuing medication. Moreover, to examine paclitaxel (DHP23002) accumulation in the tumor tissue, the amount of paclitaxel in tumor/blood was quantified using liquid chromatography with quadruple-TOF mass spectrometry.

**Results:**

In the mouse pharmacokinetic study, oral Taxol^®^ showed a negligible absorption, whereas DHP23002 showed a high absorption rate dependent on dosage, with a bioavailability of approximately 40% at a dose of 62.5 mg/kg. In efficacy-related studies, DHP23002 administration at a dose of 25, 62.5, or 125 mg/kg on alternate days for 3 weeks showed a superior tumor inhibitory effect of 80%, 92%, and 97% in a xenograft mouse model, respectively, after 7 weeks. Paclitaxel accumulation in tumors persisted for >24 h in mice, when orally administered once at doses of 25, 62.5, and 125 mg/kg DHP23002.

**Conclusion:**

Oral chemotherapy with DHP23002 showed excellent absorption in animals owing to a strong antitumor activity in a pancreatic cancer mouse model. This demonstrates that paclitaxel is largely distributed and persists for a prolonged period at the tumor site owing to oral DHP23002 administration.

## Introduction

Globally, pancreatic cancer has been reported as the twelfth most common cancer and is the seventh leading cause of cancer-associated mortality. More than 330,000 people die from pancreatic cancer, and almost the same number of new cases are reported annually [1–3]. Because approximately 80% of patients with pancreatic cancer have progressive or metastatic disease, patients have a very poor prognosis of ≤6 months, and the estimated 5-year survival rate is approximately 6% [4].

Over the past few decades, small chemotherapeutic agents such as 5-FU or gemcitabine and drug combinations have been tested for treating progressive pancreatic cancer [5]. In 2010, a new chemotherapy for advanced pancreatic cancer, called FOLFIRINOX, was introduced, which increased the survival rate by several months in clinical trials as opposed to the conventional chemotherapy [6]. Although FOLFIRINOX, an intensive cytotoxic regimen comprising 5-fluorouracil, leucovorin, irinotecan, and oxaliplatin, was an attractive strategy for pancreatic cancer therapy, it caused toxicity or was associated with several adverse side effects, including febrile neutropenia, fatigue, diarrhea, and peripheral neuropathy [7].

Because taxane can enhance microtubule assembly and inhibit tubulin depolymerization and consequently block cell proliferation, use of docetaxel or paclitaxel as a single agent or in combination with other chemotherapeutics has been proposed for pancreatic cancer. Poor solubility problem of taxane could be resolved by developing a polyoxyethylated castor oil solvent (Cremophor EL) formulation, which can be intravenously administered (*Cre*-paclitaxel; Taxol^®^) [8]. However, Cremophor EL-induced toxicity warranted the administration of steroids and antihistamines prior to intravenous (i.v.) infusion for reducing the adverse side effects, such as bronchospasm, hypotension, neuro- and nephrotoxicity, and anaphylactic reactions, caused by C*re*-paclitaxel formulation. Moreover, The Cremophor EL solvent also altered the pharmacology of paclitaxel, thereby affecting its toxicity profile and anticancer efficacy [9–10]. Thus, numerous strategies have been developed to better solubilize and improve the pharmacology of paclitaxel, including the use of albumin nanoparticles, emulsions, and liposomes. Recently, the Metastatic Pancreatic Adenocarcinoma Clinical Trial (MPACT), which used a combination therapy of nab-paclitaxel, Abraxane^®^, and gemcitabine, revealed an increased survival rate of patients compared with the survival rate of patients who received FOLFIRINOX [11–12]. Additionally, compared with FOLFIRINOX, MPACT had a more acceptable and manageable toxicity level and adverse side effects, such as neutropenia and neuropathy.

Because the oral paclitaxel formulation can be conveniently administered and provides timing and location flexibility for patients with pancreatic cancer, the development of an oral formulation would prevent unnecessary hospitalization and hypersensitivity reactions. Moreover, as opposed to intermittent iv exposure, oral administration of paclitaxel reportedly allows continuous exposure at low and effective concentrations during the treatment period and thereby facilitates a more flexible drug administration procedure. It has been reported that continuous exposure to low paclitaxel concentrations exerts a superior anti-tumor effect by inhibiting tumor-associated angiogenesis [13].

Recently, a clinically usable, mucoadhesive, lipid-based oral paclitaxel formulation (DHP107) has been developed [13–14]. The formulation comprises edible lipids and a Food and Drug Administration-approved emulsifier and does not include toxic excipients. This study aimed to investigate DHP23002, another oral paclitaxel formulation that is slightly different in composition than DHP107, as well as compare the antitumor efficacy of DHP23002 with that of traditional i.v. Taxol^®^ *in vivo* using a xenograft mouse model of chemotherapy-sensitive BxPC-3 pancreatic cancer.

## Materials and methods

### Ethics statement

All animals including ICR and BALB/c nude mice were maintained and used in accordance with the Guidelines for the Care and Use of Laboratory Animals of the Institute of Laboratory Animal Center, Daegu-Gyeongbuk Medical Innovation Foundation. The animal studies were conducted after approval by the Institutional Reviewer Board (IRB) on the Ethics of Animal Experiments of the Daegu-Gyeongbuk Medical Innovation Foundation (approval number: DGMIF-18010902-00). All efforts were made to minimize and euthanize the pain of mice according to the end points of an IACUC-approved protocol.

### Preparation of oral paclitaxel and animals

Taxol^®^ was purchased from Bristol-Myers Squibb (BMS, USA), and paclitaxel powder for preparing DHP23002 was purchased from Samyang Bio Pharmaceutical^®^ (Daejeon, South Korea). Oral paclitaxel (DHP23002) and vehicle were prepared by Dae Hwa Pharmaceutical Co. Ltd. (Hoengseong, Korea), and their preparation method has been described in the patent [15]. All solvents and reagents were obtained from Sigma-Aldrich (St. Louis, MO, USA). ICR female mice (5–6 weeks old) and athymic BALB/c nude mice (6 weeks old) were supplied by Orient Bio Ltd. (Seongnam, Gyeonggi do, Korea) and maintained in pathogen-free conditions in the animal facility at Daegu-Gyeongbuk Medical Innovation Foundation (DGMIF).

### Pharmacokinetic study of an oral formulation, *DHP23002*

Six female ICR mice were administered with an oral formulation of paclitaxel (DHP23002) at doses of 62.5, and 125 mg/kg. Taxol^®^ (*Cre*-paclitaxel) was diluted six times with saline solution and administered via a bolus tail-vein injection at a dose of 12.5 mg/kg as a positive control. Taxol^®^ (*Cre*-paclitaxel) for oral administration was prepared by dissolving paclitaxel at a concentration of 36 mg/mL in an equivolume mixture of Cremophor EL & ethanol and diluted with saline solution; this mixture was orally administered at a dose of 62.5 mg/kg and used as another control. To determine paclitaxel concentration in plasma, a 200-μL plasma sample was mixed with 800 μL acetonitrile after centrifuging at 10,000×*g* for 10 min to recover the supernatant. The reconstituted solutions were centrifuged for 10 min at 10,000×*g* at 4°C to eliminate the proteins in plasma samples. The supernatant was transferred to a total recovery vial for analysis. LC-MS/MS analysis was performed using Xevo G2-XS (Waters Corporation, MA, USA) coupled to a Q-TOF mass spectrometer (Waters Corporation) equipped with an electrospray ionization source. The two mobile phase gradient composition was solvent A (water with 0.1% formic acid) and solvent B (acetonitrile with 0.1% formic acid). A 3-min gradient was developed from 50% to 10% A at a flow rate of 0.3 mL/min. The analytical column was a Waters BEH C18 UPLC Column (1.7 μm, 130 Å, Waters Corporation). Ion detection was performed in MS mode, and the parent ions were monitored at 854.3 *m/z*. Data acquisition was performed using TargetLinx software (Waters Corporation). The mean and standard error of the mean at each timepoint were calculated, and PK parameters were determined from the mean concentration data via a non-compartmental analysis conducted using the WinNonlin 6.4 program (Pharsight Corporation, Mountain View, CA, USA).

### Pancreatic cancer xenograft model for DHP23002 efficacy test

A luciferase-expressing human pancreatic adenocarcinoma cell line, BxPC3-Red-Fluc (Bioware^®^ Brite cell line), was purchased from Perkin Elmer. The BxPC3 cell line was maintained in RPMI-1640 supplemented with 10% fetal bovine serum (Hyclone, Logan, UT, USA) and 1% PS (Penicillin/Streptomycin). Six-week-old female, athymic nude mice (NCr-nu) were adapted for 1 week. Before cell line injection, pancreatic cancer cells were washed twice with phosphate-buffered saline (PBS), detached using 0.5% Trypsin-EDTA, harvested by centrifugation, and resuspended in Hank’s balanced salt solution (HBSS; Invitrogen, Carlsbad, CA, USA). Cell viabilities were determined by trypan blue exclusion method. For injection into nude mice, mice were anesthetized by continuous inhalation of 3% isoflurane gas for 5–10 min using an anesthesia system (Harvard Apparatus, UK). A suspension of 2.0×10^6^ cells/100 μL was subcutaneously injected on the right lower back of the mouse. The skin around the tumor was swabbed using a combination of betadine and alcohol for minimizing the risk of bacterial infection. Tumor cell-implanted animals were placed in a separate individual cage and then returned to IVIS cages (Individually ventilated cage, Techniplast, Italy) when they regained consciousness after anesthesia.

The experiment was performed to compare the therapeutic efficacy of Taxol^®^ (BMS) and DHP23002 (Dae Hwa Pharmaceutical Co. Ltd.). To evaluate the efficacy of DHP23002, eight female athymic nude mice received 0, 25, 62.5, and 125 mg/kg DHP23002 orally on alternate days for 3 weeks. Oral administration of DHP23002 vehicle (DHP system) was used as negative control; Taxol^®^ intravenous injections at 10 mg/kg were administered weekly as positive control. Mice (8 per treatment group) were randomly assigned to receive one of five treatments: (1) 5 mL/kg of vehicle administered once every other day (control group); (2) 10 mL/kg of Taxol^®^ administered intravenously once weekly; (3) 1 mL/kg of DHP23002 administered once on alternate days (4) 2.5 mL/kg of DHP23002 administered once on alternate days (5) 5 mL/kg of DHP23002 administered once on alternate days. Treatments were continued for 3 weeks, and the tumor size of each group was observed for 48 days (generally 7 weeks). Tumor volume was measured every week after inoculation and calculated as follows: tumor volume = short length×short length×long length×0.5. After observation, all mice were imaged using IVIS-spectrum-CT (Calipers, USA) after injection of luciferin for luciferase activation (S1 Fig).

### Paclitaxel accumulation in BxPC-3 tumor-bearing mice

To evaluate the amount of paclitaxel in tumor or blood after the first administration of paclitaxel formulation via i.v. or oral routes, tumor-bearing mice were prepared. BxPC-3 cells at a concentration of 2.0×10^6^ cells/100 μL was subcutaneously injected on the right lower back of mice, and those mice were cared for until the tumor volume became ≥200 mm^3^. The experiment was performed to evaluate paclitaxel accumulation in tumors after i.v. administration of Taxol^®^ (10 mg/kg) and oral administration of DHP23002 (25, 62.5, and 125 mg/kg) and to monitor the amount of paclitaxel in blood after administration. Taxol^®^ or DHP23002 was administered at the same dose as the abovementioned tumor growth inhibition experiment. At 6, 24, and 48 h after the administration of each formulation, mice were euthanized by CO_2_ asphyxiation and tumor homogenates and blood plasma were collected for further analysis. Tumor tissues were homogenized in four volumes of PBS and centrifuged for 10 min at 10,000×g. Two hundred microliters of supernatant fraction was mixed with 800 μL acetonitrile and filtered through a 0.2-μm PTFE syringe filter (Whatman). Acetonitrile extracts of tumor tissue and blood were analyzed via LC-MS/MS analysis (Xevo G2-XS Q-TOF, Waters Corporation). The composition of the two mobile phase gradients was solvent A (water with 0.1% formic acid) and solvent B (acetonitrile with 0.1% formic acid). Ion detection was performed in MS mode, and parent ions were monitored at *m/z* 854.3. Data acquisition was performed using TargetLinx software (Waters Corporation).

### Action mechanism study of DHP23002 via immunohistochemistry and flow cytometry analysis

Immunohistochemical analysis was performed to examine the localization of β-tubulin proteins in untreated, Taxol^®^-, or DHP23002-treated cells. Briefly, 2×10^4^ cells were grown on a chamber slide^™^ (Lab-Tek II; Thermo Fisher Scientific, MA, USA). After the cells attained 70%–80% confluence, fresh RPMI 1640 (GIBCO, Thermo Fisher Scientific) medium containing 0.5% fetal bovine serum and antibiotics was added to each dish and subsequently treated with vehicle, Taxol^®^, and DHP23002 for 4 h. After a 4-h incubation period, cells were washed with PBS and fixed with 4% paraformaldehyde solution for 30 min at 4°C. After fixing cells, the chamber slide was washed again with PBS and incubated for 30 min with 3% BSA containing 0.2% triton X-100 (blocking solution). After washing with PBS, cells were further incubated for 1 h at 25°C with beta-tubulin antibody (dilution, 1:100; Invitrogen) in 3% BSA containing 0.2% triton X-100. Unbound antibodies were washed with PBS three times; the bound antibodies were stained for 0.5 h at 25°C with an anti-rabbit secondary antibody conjugated to fluorescein isothiocyanate (FITC) (dilution, 1:500; Thermofisher Scientific, Eugene, OR) by incubating them together in 3% BSA containing 0.2% triton X-100, which was prepared in PBS. After washing, a drop of 4ʹ,6-diamidino-2-phenylindole (300 nM in mounting solution) was added. Slides were viewed under K-1 imaging upright microscope (K-1 FLUO confocal microscopy, Korea) system using different filters.

Apoptosis was assessed by staining with annexin-V and propidium iodide (PI), which is a well-known method for apoptosis detection. BxPC-3 cells (1×10^6^/mL) were seeded and incubated for 48 h. Taxol^®^-treated or DHP23002-treated BxPC-3 cells were collected after 24-h treatment. Apoptosis was detected using an FITC-Annexin V/propidium iodide (PI) kit (EzWay Annexin V-FITC Apoptosis Detection Kit, Koma Biotech, South Korea), according to the manufacturer’s instructions. In brief, the cells were washed with cold PBS and subsequently stained with a combination of 1.25 μL FITC-Annexin V and 10 μL PI for each 100-μL cell suspension and incubated at room temperature for 15 min. Apoptotic cells were immediately analyzed using a flow cytometer (Beckman Coulter, USA). The apoptosis of samples was given as the sum of early and late apoptosis.

### Statistical analysis

Statistical analysis was performed using Sigma Plot v.10.0 (Systat Software Inc.). Paired T-test was used to determine the statistical significance of the results. A P-value, 0.05 was considered statistically significant and at least 5~8 mice per group were used for each *in vivo* experiment.

## Results

### Pharmacokinetic profile of DHP23002

**Fig 1:**
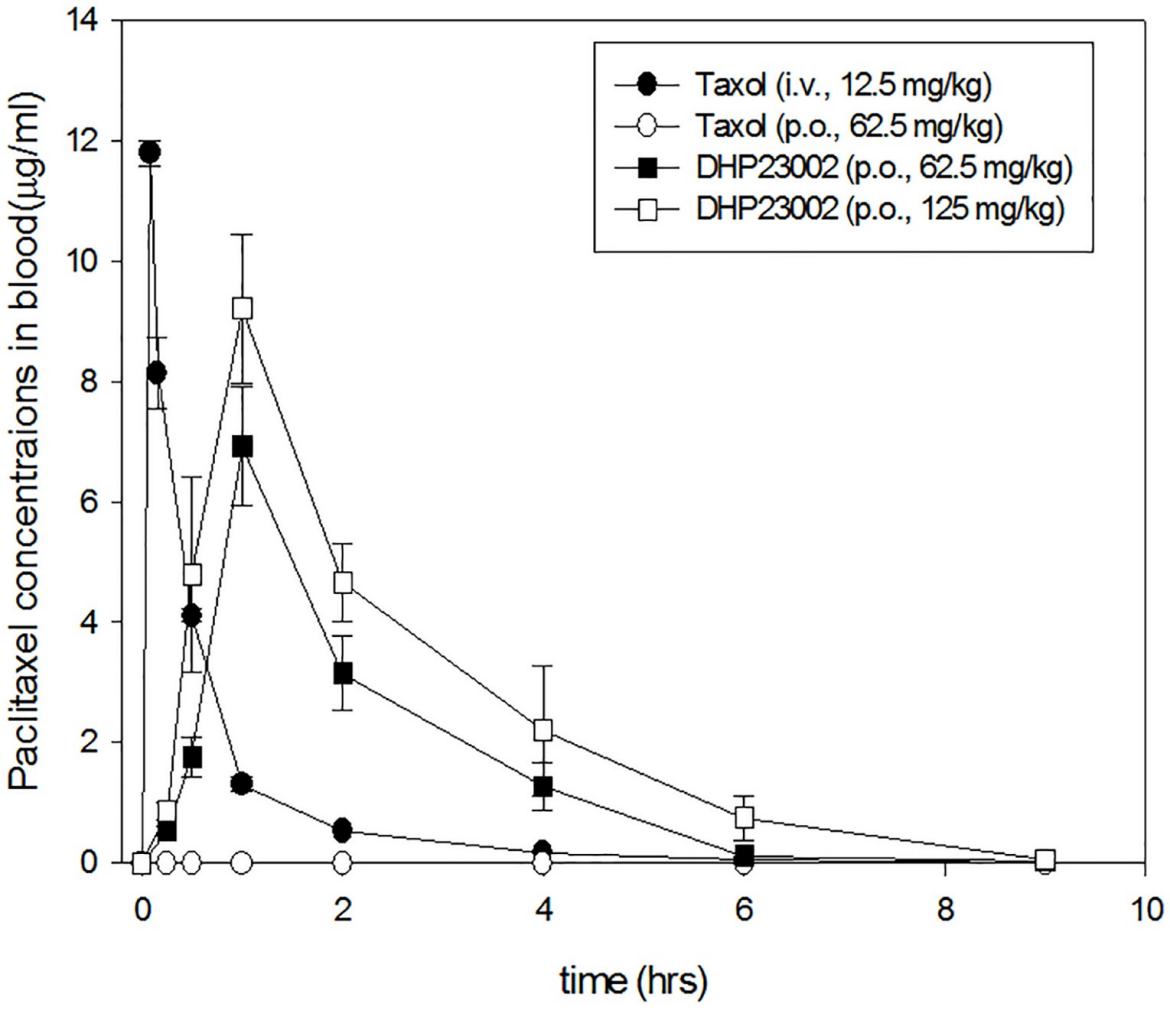
The pharmacokinetic profiles of paclitaxel in mice after oral administration of Taxol^®^ or DHP23002.

Plasma concentration–time profiles for the oral paclitaxel, DHP 23002, were obtained using different paclitaxel doses to evaluate whether it is an orally active drug. DHP23002 formulation consisted of three safe lipid ingredients, one surfactant, and paclitaxel at a concentration of 25 mg/mL. Its solubility was comparable to any commercially available paclitaxel formulation. As shown in Fig 1 and Table 1, the oral administration of paclitaxel-formulated with Cremophor EL (Taxol^®^) resulted in a very low bioavailability of approximately 0%. However, the mean AUC values of DHP23002, which was developed for enhancing the oral absorption of paclitaxel, at doses of 62.5 mg/kg and 125 mg/kg was approximately 13.63±2.7 μg∙h/mL and 22.26±5.68 μg∙h/mL, respectively. Moreover, C_max_ of DHP23002 at 62.5 mg/kg and 125 mg/kg was approximately 6.9±0.98 μg/mL and 9.2±1.23 μg/mL, respectively.

**Table 1:** Pharmacokinetic profiles of paclitaxel formulations in mice.

Formulations	Paclitaxel Dose (mg/kg)	T_max_ (h)	C_max_ (μg/mL)	Half life (h)	AUC (μg∙h /mL)	BA (%)	Animal model
Taxol^®^	12.5 (i.v.)	0.083	11.79±0.21	0.81±0.002	6.64±0.33	100	Mice
Paclitaxel via oral route	62.5 (p.o.)	-	-	-	-	-	Mice
DHP23002	62.5 (p.o.)	1	6.92±0.98	1.50±0.073	13.63±2.70	41.0±8.12	Mice
	125 (p.o.)	1	9.2±1.23	1.64±0.134	22.26±5.68	33.5±8.43	Mice

### Evaluation of the therapeutic effect of DHP23002 chemotherapy

**Fig 2:**
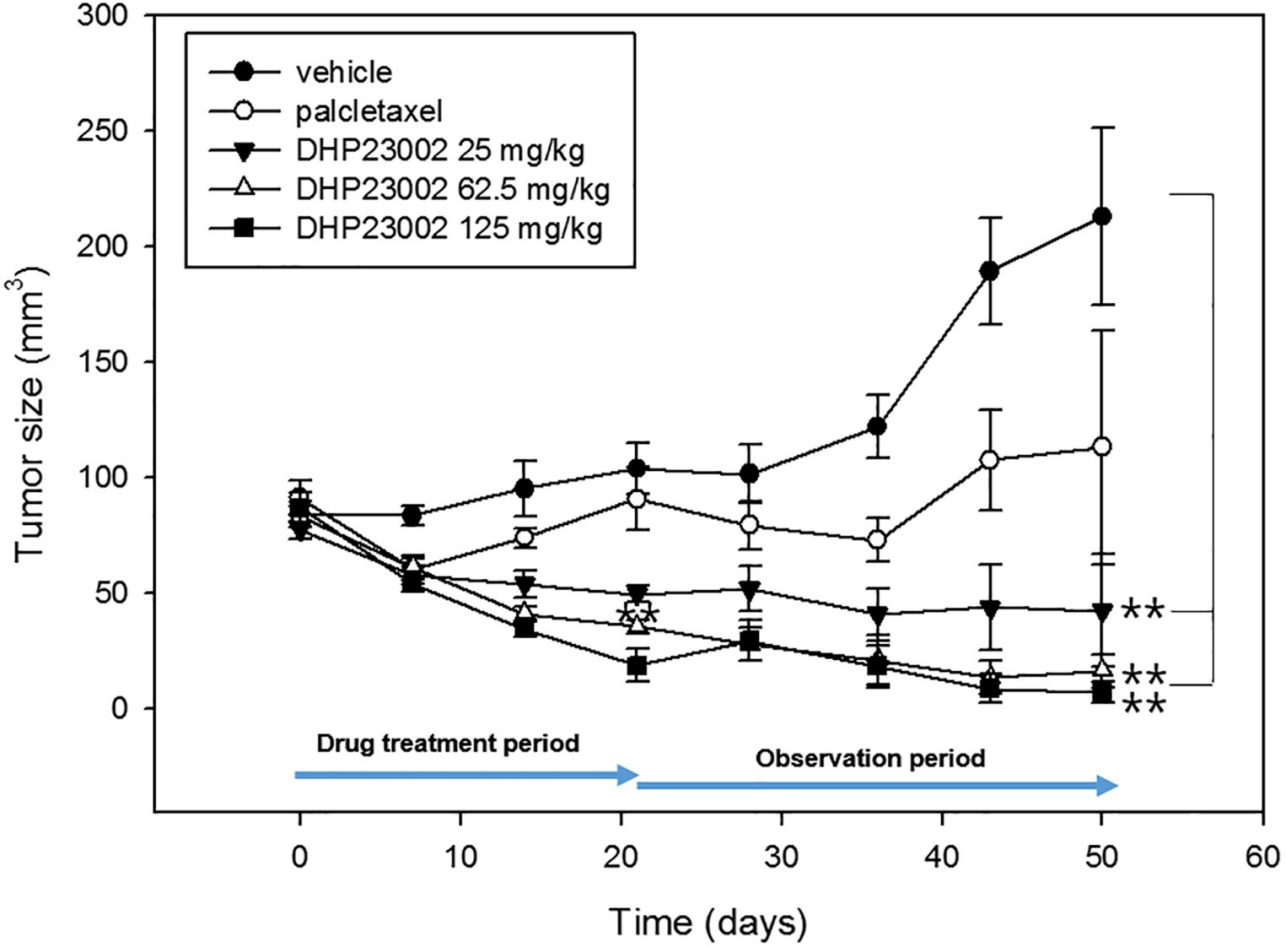
Tumor-bearing mice were prepared using the pancreatic cancer cell line, BxPC-3-red-luc, to observe tumor retardation effect induced by oral DHP23002. As the negative control, the formulation vehicle without paclitaxel (●) and DHP23002 was orally administered at doses of 25 (▼), 62.5 (Δ), and 125 mg/kg (■) every day for 3 weeks. As the positive control, Taxol^®^ (○, 10mg/kg) was intravenously administered (Mean±SEM). 0.05*>P, 0.01**>P, as compared with the vehicle treated control.

To monitor the effect of the oral paclitaxel DHP23002 on tumor growth, we explored tumor retardation effect on tumor-bearing mice. As shown in Fig 2, at 7 weeks after oral administration, we observed that treatment of tumor-bearing mice with DHP23002 at doses of 25 mg/kg (42.4±24.5 mm^3^, p<0.01), 62.5 mg/kg (16.5±7.1 mm^3^, p<0.01), and 125 mg/kg (7.2±4.7 mm^3^, p<0.01) not only decelerated tumor growth but also reduced tumor size as opposed to the treatment of control mice with vehicle (213±38.6 mm^3^). Moreover, the average tumor volume in mice 7 weeks after i.v. administration of Taxol^®^ was 113.1±50.4 mm^3^.

Tumor reduction was observed at 7 days in case of both Taxol^®^ and oral DHP23002 treatment; it is interesting to note that administration of oral DHP23002 for the first 3 weeks affected tumor growth for 7 weeks. As shown in Fig 2, after discontinuation, the tumor size in the oral DHP23002 group continued to decrease, but in the i.v. Taxol^®^ group, the tumors size increased again at 7 weeks. Further, the minimum dose of DHP23002 used in this study to inhibit tumor growth was 25 mg/kg; this led to a significant inhibition in tumor growth. After 3 weeks of drug administration or 4 weeks after drug discontinuation, the tumor size of group treated with 25 mg/kg DHP23002 decreased by 52.3% and 81% of tumor size, respectively, when compared with the vehicle control.

### Paclitaxel accumulation in tumors after administration of Taxol^®^ and DHP23002

**Fig 3:**
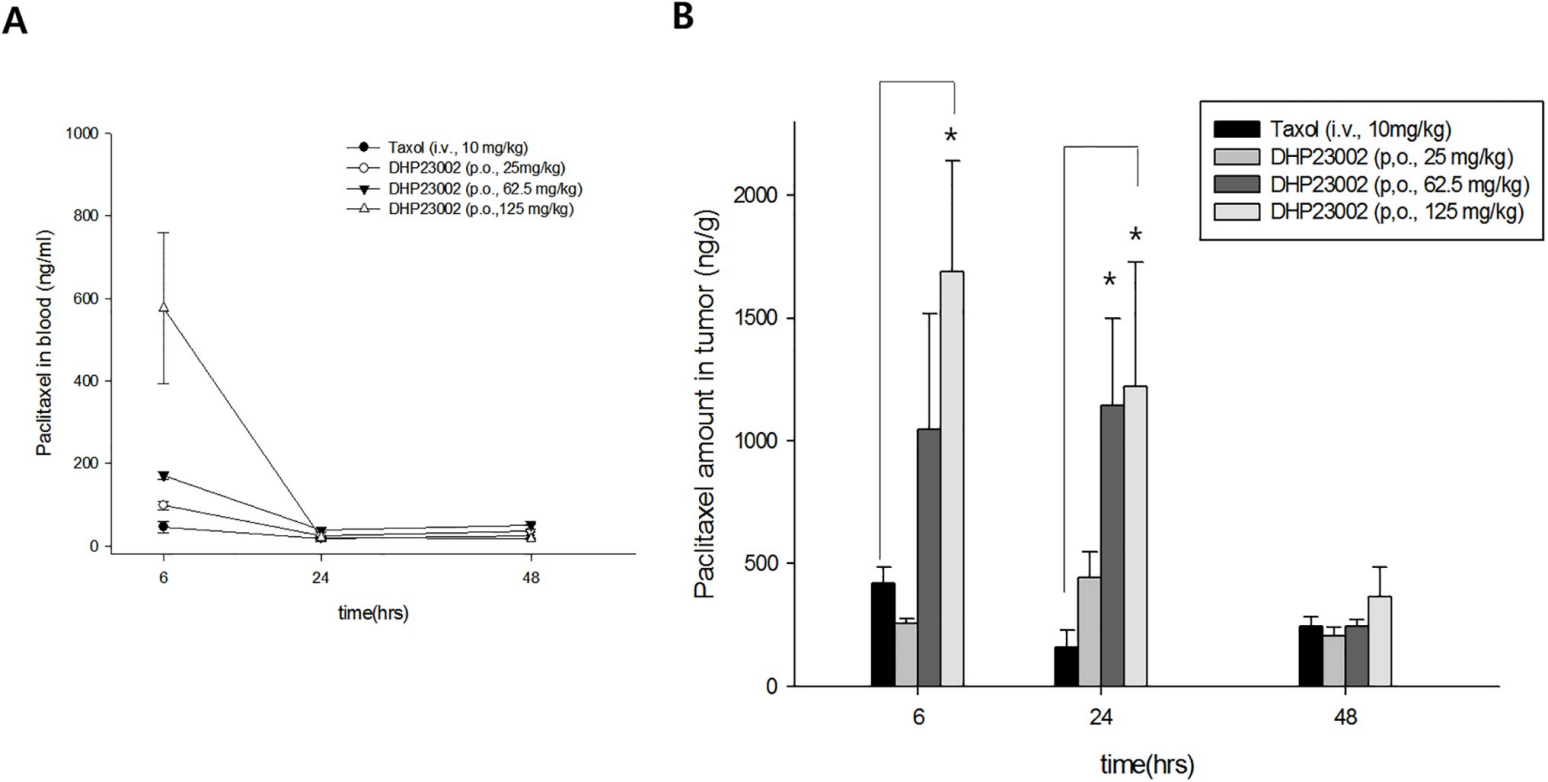
Quantification of paclitaxel in blood or tumors of pancreatic tumor-bearing mice after the administration of Taxol^®^ or DHP23002. To observe paclitaxel accumulation in tumors after the administration of 10 mg/kg Taxol^®^ or 25, 62.5, and 125 mg/kg DHP23002, the amount of paclitaxel in blood (A) and tumors (B) at 6, 24, and 48 h after administration was analyzed using LC-MS/MS. (mean±SEM), 0.05*>P.

The findings of the PK study on intravenously administered Taxol^®^ revealed that at 6 h after Taxol^®^ intravenous injection, paclitaxel concentration in blood was low at 46.6±13.8 ng/mL, followed by a general elimination pattern until 48 h. Contrastingly, PK studies of oral DHP23002 revealed blood concentrations of 98.5±10.7, 170.8±9.1, and 577.0±182.8 ng/mL for doses of 25, 62.5, and 125 mg/kg, respectively, at 6 h after administration. However, at 24 and 48 h after administration, the concentration of DHP23002 in blood for each dose was reduced to nearly the same concentration of intravenously administered Taxol^®^ (10 mg/kg) in blood (Fig 3A).

Paclitaxel amount in tumors after Taxol^®^ i.v. injection was approximately 421.0±63.9 ng/g at 6 h, and low paclitaxel levels of 160.7±67.8 and 244.2±40.6 ng/g were detected at 24 and 48 h. However, at 6 h, oral DHP23002 administration led to high accumulation of paclitaxel in tumors at concentrations of 255.7±21.1, 1047.5±472, and 1689.8±450.6 ng/g when administered at doses of 25, 62.5, and 125 mg/kg, respectively. (Fig 3B). Remarkably, a significant amount of paclitaxel was detected in tumors even at 24 h, i.e., 441.7±107.7, 1143.1±355.7, and 1220.8±508.6 ng/g, in 25, 62.5, and 125 mg/kg oral DHP23002 dose groups, respectively.

These results demonstrate that oral administration of DHP23002 can facilitate accumulation and maintenance of high paclitaxel concentrations in tumors for more than 1 day. Comparison between paclitaxel concentrations in tumors and blood revealed that paclitaxel accumulation in tumors was high at 6 and 24 h, even though the drug was present at very low concentrations in blood at 24 h.

### Flow cytometric *and* immunohistochemical analyses of the mechanism underlying the effect of DHP23002 on BxPC-3 cancer cells

**Fig 4:**
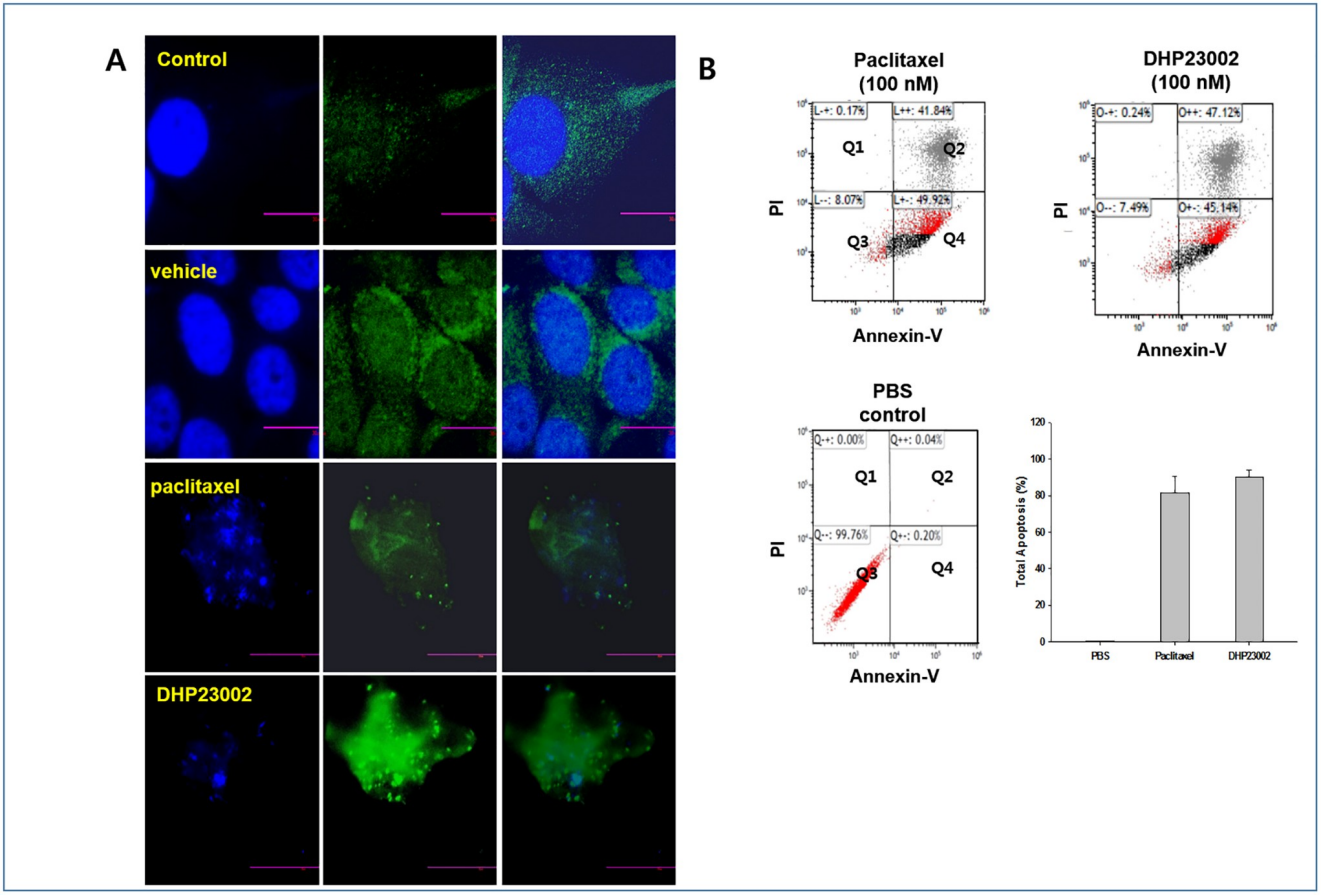
Images of fluorescently-labeled beta-tubulin in BxPC-3 cells treated with paclitaxel or DHP23002. BxPC-3 cells were treated with 100 nM paclitaxel or DHP23002 for 4 h or with DHP23002 vehicle as a control. Microtubule structure (green) was visualized by fluorescent labeling using anti-β-tubulin rabbit primary antibody followed by staining with anti-rabbit-fluorescein isothiocyanate, as described in the "Materials and Methods". Nuclei are counterstained using 300 nM 4′,6-diamidino-2-phenylindole (blue). Apoptosis assay conducted via FACS for BxPC-3 cells treated with Taxol^®^ or DHP23002. BxPC-3 cells were harvested and resuspended in binding buffer after treatment with paclitaxel (100 nM) for 24 h. Cell apoptosis was subsequently analyzed using flow cytometry. In the right lower panel in Fig 4B, Total apoptosis (%) was defined as the sum of percentages of early and late apoptotic cells. PI, propidium iodide.

Although the mechanism of paclitaxel in the killing of cancer cells is already known, immunohistochemistry and flow cytometry were performed to observe whether DHP23002, which is a paclitaxel-based formulation, also has the same mechanism of action against BxPC-3 cancer cells. First, we tested whether both Taxol^®^ and DHP23002 had an effect on microtubule polymerization using an immunofluorescence assay accompanied by visualization under a confocal microscope. As shown in Fig 4A, we observed an abnormal dense microtubule distribution around nuclear fragments as well as inhibition of microtubule depolymerization by 100 nM paclitaxel and DHP23002 (formulated using 100 nM paclitaxel). These results indicated that both Taxol^®^ and DHP23002 had a similar action mechanism on BxPC-3 cells. We also performed Annexin-V-FITC/PI staining to evaluate BxPC-3 cell death after Taxol^®^ or DHP23002 treatment. Based on flow cytometry results, we found that 100 nM Taxol^®^ or DHP23002 treatment significantly increased the percentage of apoptotic cells (early and late apoptosis) as opposed to treatment with vehicle in the control group (Fig 4B). The percentage of apoptotic cells upon treatment with 100 μM of Taxol^®^ or DHP23002 in BXPC-3 cells were found to be about 81.5% and 90.2% after 24 h incubation (Fig 2). Our results showed the similar serious apoptosis occurred in both Taxol^®^ and DHP23002-treated groups when the same amount of paclitaxel was used in the formulations.

## Discussion

This study aimed to investigate the antitumor efficacy of DHP23002, a new oral paclitaxel formulation, in comparison with that of traditional i.v. Taxol^®^ using a xenograft mouse model of BxPC-3 pancreatic cancer.

As mentioned previously, application of paclitaxel in clinical settings is limited by its insolubility in water. Thus, Taxol^®^, a paclitaxel formulation containing a mixture of ethanol and Cremophor EL which is used to stabilize a nonpolar paclitaxel emulsion in water, has been developed and applied in clinical settings. However, Cremophor EL causes drug-related hypersensitivity reactions including dyspnea, tachycardia, hypotension, angioedema, and generalized urticarial reaction in 2%–4% of patients receiving Taxol^®^ [8]. The issues associated with conventional Taxol^®^ have driven the need for development of new oral formulation with notable advantages, such as convenience, flexibility of timing and location, and minimal risk of infection, as opposed to the drug formulations for intravenous or intraperitoneal administration. Recently, metronomic DHP107, a novel oral form of paclitaxel, was introduced as an alternative of Taxol^®^. DHP107, consisting of mono- and tri-glycerides as well as polysorbate 80, is an effective lipid-based oral paclitaxel administered without Cremophor EL and has high bioavailability without P-glycoprotein inhibitors [13–14]. The development of an oral active form of paclitaxel will not only increase efficacy but also reduce side effects. Furthermore, metronomic therapy will be possible because cytotoxic chemotherapeutic drugs can be administered at more frequent intervals without prolonging rest periods.

Here, we used DHP23002, which has subsequently been developed from DHP107 and consists of an oleoylglycerol complex, a polyoxyl glyceryl fatty acid ester, medium-chain triglycerides, and a surfactant. The most important advantage of DHP23002 is that it can be administered at high doses. The toxicity of paclitaxel remains a point of concern owing to a narrow therapeutic window and inter-individual PK variability that causes unpredictable toxicity. Currently, for cancer therapy, paclitaxel is injected at an i.v. dose of 175 mg/m^2^ over a 3-h infusion period, every 3 weeks [16]. Considering the maximum tolerated dose of Taxol^®^, administration of a high dose of i.v. Taxol^®^ on a weekly basis to the mice in this study was not permitted. Even when the mice were treated with DHP23002 at a higher dose than that of i.v. Taxol^®^, which is an advantage of oral formulations, paclitaxel level in blood did not increase abruptly. In particular, DHP23002 can be orally administered at high doses, whereas high doses of i.v. Taxol^®^ injection cannot be administered all at once.

As shown in Fig 2, DHP23002 is an oral, active lipid-based formulation having strong anti-tumor effects in a pancreatic cancer mouse model (BxPC-3). When DHP23002 was administered at a dose of 25 mg / kg once every two days for 21 days, it inhibited about 52.3% tumor growth compared to the vehicle-administered group during the administration period, but 81% tumor suppression was observed 4 weeks after DHP23002 discontinuation.

In particular, at 7 weeks, tumors were almost eliminated in three mice in the group receiving 62.5 mg/kg DHP23002 and in five mice in the group receiving 125 mg/kg DHP23002 (S1 Fig). Tumor suppression outcomes for 10 mg/kg Taxol^®^ or 125 mg/kg DHP23002 shown in Fig 2 overlap with the outcomes of drug accumulation in tumor shown in Fig 3(B). Significantly, tumor growth continued to decrease, even after 3 weeks of drug discontinuation. These results showed that early treatment of cancer with DHP23002 would have long-term effects on tumor growth, and thereby suggest that paclitaxel formulated with DHP23002 persisted within the tumors for a prolonged period and was only slowly eliminated. The reason for the paclitaxel persistence in tumors remains unclear; however, based on the mechanism of DHP107, we can make a few assumptions regarding DHP23002. DHP23002 can prolong the release of paclitaxel in the intestine to enhance the absorption and stability of the drug in the tumor and can evade the action of P-glycoproteins [17–18]. In a PK experiment conducted on large animals, DHP23002 showed similar PK patterns, and it was confirmed that an effective paclitaxel concentration was maintained in blood for a prolonged period even when low doses were administered (S2 Fig). Further research is warranted to understand how paclitaxel is slowly absorbed through oral DHP23002 and remains in the tumor for an extended period of time.

Moreover, the composition of DHP23002 has two more advantages compared with DHP107. First, it does not solidify at 4°C as opposed to DHP107, and second, *DHP23002* can contain 25 mg paclitaxel in a 1-mL formulation, whereas DHP107 can only contain 10 mg paclitaxel in a 1-mL formulation. Therefore, it has been suggested that oral capsule formulation of DHP23002 may allow the implementation of various administration schedules. Research on DHP23002 is ongoing and has shown some advantages and disadvantages of the drug; therefore, currently, it is difficult to predict whether DHP23002 is better than DHP107.

While it is true that paclitaxel solubility and toxicity issues are limited to clinical use, DHP23002 certainly addresses these two problems in the development of a new oral formulation of paclitaxel. Specifically, such an oral paclitaxel formulation is well absorbed when orally administered up to a dose of 25 mg/mL. In addition to being well absorbed into the intestinal tract, the drug concentration can be increased directly in the formulation owing to its oral intake; this, in turn, can alleviate inconvenience and the pain suffered by patients owing to infusion injections. Furthermore, a single toxicity reported that the approximate lethal dose of DHP23002 was >625 mg/kg for both sexes, and thus, is a safer drug formulation than i.v. Taxol^®^ injections, which has an LD50 value of 12.5 mg/kg. The former will prove to be a useful drug formulation in oral clinical trials because it facilitates delivery of high doses without the need for continuous administration, and is gradually absorbed in and slowly eliminated from cancer tissues.

This study showed that the newly developed oral paclitaxel DHP23002 combined with a high concentration of paclitaxel can reduce the required number of doses and maintain an effective concentration in blood continuously for several days. Although this study has demonstrated the potential use of DHP23002 as a first-line chemotherapeutic agent in a xenograft mouse model of pancreatic cancer, future studies are needed to establish the maximum tolerated dose of DHP23002 in rodent and non-rodent models.

## Supporting information

S1 Fig Observation of tumor retardation by IVIS-spec-CT. (DOCX)

S2 Fig Pharmacokinetic study of oral paclitaxel in beagle dogs. (DOCX)
