# Supplementary material for: DHP23002 as a next generation oral paclitaxel formulation for pancreatic cancer therapy
Source: PLoS One. 2019 Nov 19;14(11):e0225095. doi: 10.1371/journal.pone.0225095 (PMC6863550; doi:10.1371/journal.pone.0225095)
Supplement: S1 Fig — (DOCX) [file pone.0225095.s001.docx]

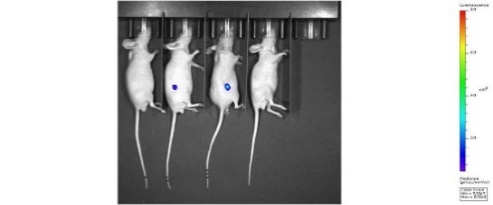

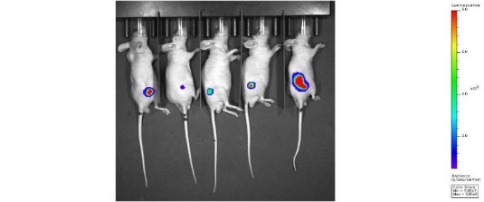

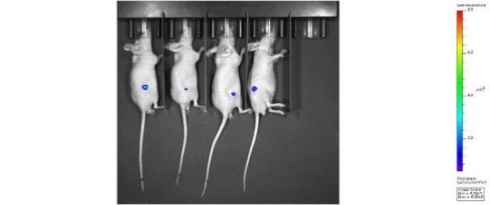

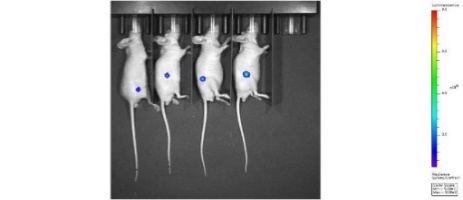

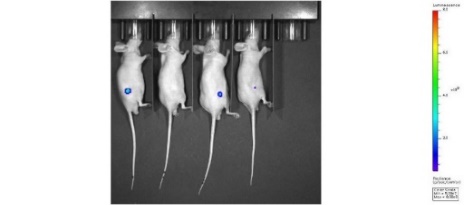

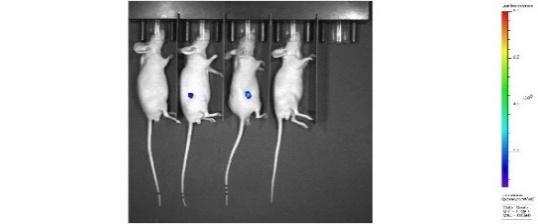


**Taxol^®^ (iv)**

**Vehicle**

**DHP23002 25 mg/kg**

**DHP23002 62.5 mg/kg**

**DHP23002 125 mg/kg**

**S1 Fig. Observation of tumor retardation by IVIS-spec-CT**

Tumor retardation experiment in the Figure 2, the growth of tumors in five randomly selected mice in each group was observed via IVIS® Spectrum *in Vivo* Imaging System (Calipers, USA), because of the bioluminescent tumors on the back of the mice. In fact, we were able to observe a significant decrease in tumor bioluminescence in mice treated with *DHP23002* and in some of them the luminescence was almost gone.
