# Supplementary material for: DHP23002 as a next generation oral paclitaxel formulation for pancreatic cancer therapy
Source: PLoS One. 2019 Nov 19;14(11):e0225095. doi: 10.1371/journal.pone.0225095 (PMC6863550; doi:10.1371/journal.pone.0225095)
Supplement: S2 Fig — (DOCX) [file pone.0225095.s002.docx]

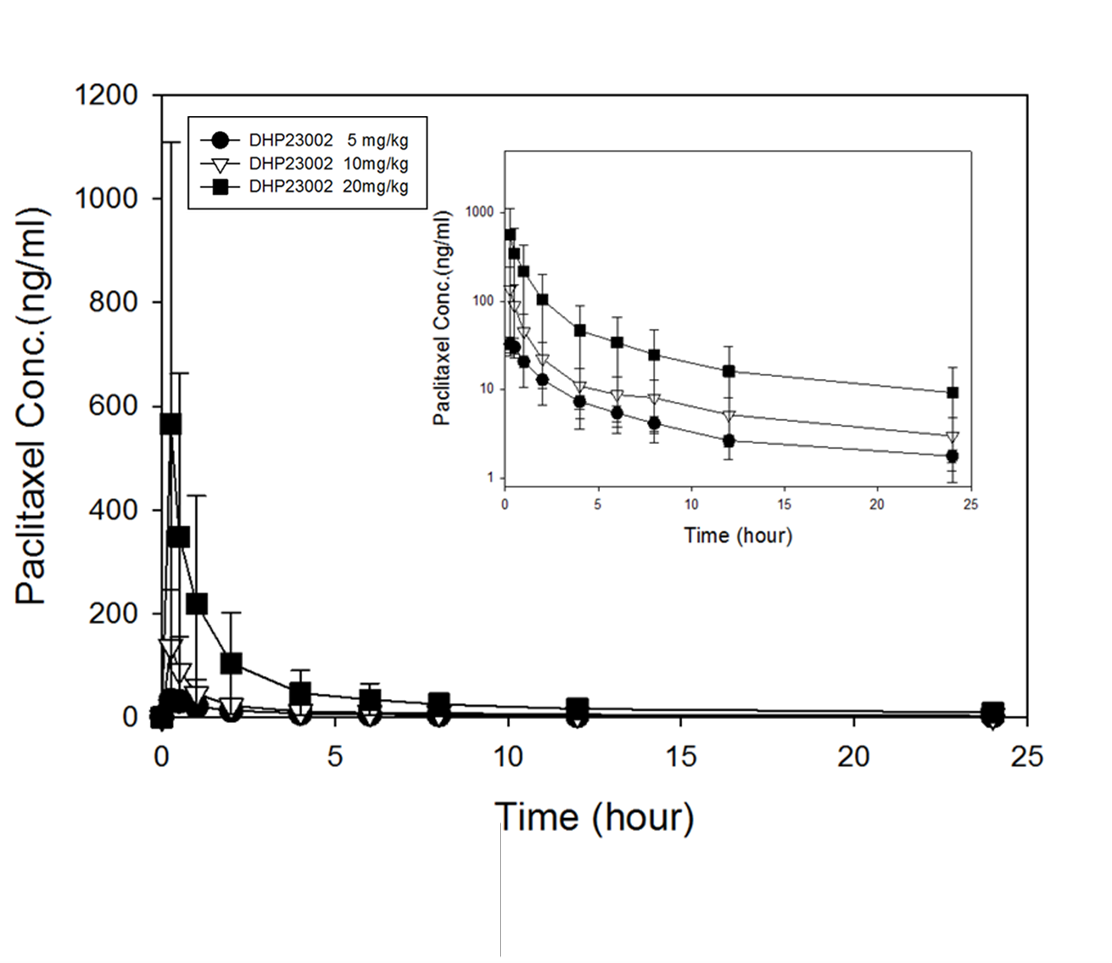


**S2 Fig. Pharmacokinetic study of oral paclitaxel in beagle dogs.**

The pharmacokinetic profiles of paclitaxel in beagle dogs (n=3) after oral administration of DHP23002. Pharmacokinetic data in beagles clearly showed that paclitaxel is absorbed very slowly even after 24 hrs.
